# Deregulation of PPARβ/δ target genes in tumor-associated macrophages by fatty acid ligands in the ovarian cancer microenvironment

**DOI:** 10.18632/oncotarget.3826

**Published:** 2015-04-15

**Authors:** Tim Schumann, Till Adhikary, Annika Wortmann, Florian Finkernagel, Sonja Lieber, Evelyn Schnitzer, Nathalie Legrand, Yvonne Schober, W. Andreas Nockher, Philipp M. Toth, Wibke E. Diederich, Andrea Nist, Thorsten Stiewe, Uwe Wagner, Silke Reinartz, Sabine Müller-Brüsselbach, Rolf Müller

**Affiliations:** ^1^ Institute of Molecular Biology and Tumor Research (IMT), Philipps University, Marburg, Germany; ^2^ Metabolomics Core Facility and Institute of Laboratory Medicine and Pathobiochemistry, Philipps University, Marburg, Germany; ^3^ Medicinal Chemistry Core Facility and Institute of Pharmaceutical Chemistry, Philipps University, Marburg, Germany; ^4^ Genomics Core Facility, Philipps University, Marburg, Germany; ^5^ Clinic for Gynecology, Gynecological Oncology and Gynecological Endocrinology, Center for Tumor Biology and Immunology (ZTI), Philipps University, Marburg, Germany

**Keywords:** PPARβ/δ, ANGPTL4, ovarian carcinoma, tumor-associated macrophages, linoleic acid

## Abstract

The nuclear receptor peroxisome proliferator-activated receptor β/δ (PPARβ/δ) is a lipid ligand-inducible transcription factor associated with macrophage polarization. However, its function in tumor-associated macrophages (TAMs) has not been investigated to date. Here, we report the PPARβ/δ-regulated transcriptome and cistrome for TAMs from ovarian carcinoma patients. Comparison with monocyte-derived macrophages shows that the vast majority of direct PPARβ/δ target genes are upregulated in TAMs and largely refractory to synthetic agonists, but repressible by inverse agonists. Besides genes with metabolic functions, these include cell type-selective genes associated with immune regulation and tumor progression, e.g., *LRP5, CD300A, MAP3K8 and ANGPTL4*. This deregulation is not due to increased expression of PPARβ/δ or its enhanced recruitment to target genes. Instead, lipidomic analysis of malignancy-associated ascites revealed high concentrations of polyunsaturated fatty acids, in particular linoleic acid, acting as potent PPARβ/δ agonists in macrophages. These fatty acid ligands accumulate in lipid droplets in TAMs, thereby providing a reservoir of PPARβ/δ ligands. These observations suggest that the deregulation of PPARβ/δ target genes by ligands of the tumor microenvironment contributes to the pro-tumorigenic polarization of ovarian carcinoma TAMs. This conclusion is supported by the association of high *ANGPTL4* expression with a shorter relapse-free survival in serous ovarian carcinoma.

## INTRODUCTION

Macrophages of the tumor microenvironment play a pivotal role in promoting the growth, invasion, metastazation and therapy resistance of malignant tumors, as suggested by the correlation of disease progression with macrophage density in different types of human cancer and shown in mouse tumor models [1, 2]. Under the influence of chemokines, cytokines and growth factors secreted by tumor cells and other host-derived cells, monocytes are recruited from the circulation and differentiate into tumor-associated macrophages (TAMs) that are programmed to promote tumor progression [3-5]. Macrophages react to their microenvironment with an extreme plasticity [6], resulting in highly diverse phenotypes, with pro-inflammatory “M1” and anti-inflammatory “M2” macrophages [4] as the extremes. Macrophages can also adopt mixed-polarization phenotypes with properties of both M1 and M2 cells [6], TAMs being a prominent example [4, 5, 7, 8].

Macrophage polarization is regulated by a plethora of signaling molecules and transcriptional regulators. These include the nuclear receptor proliferator-activated receptor β/δ (PPARβ/δ), a ligand-inducible transcription factor with established functions in intermediary metabolism and immune regulation [9, 10]. The latter has been documented in several reports addressing the role of PPARβ/δ in inflammatory responses of the skin [11, 12] and the M2-like polarization of macrophages in adipose tissue and liver [13, 14]. PPARβ/δ has also been implicated in tumorigenesis in a number of studies with conflicting results [15], which may be due to divergent functions of the receptor in tumor cells and tumor-associated host cells as well as differences in the experimental models used (mouse strains, synthetic ligands).

PPARβ/δ binds to PPAR response elements (PPREs) at its target genes as a heterodimer with a retinoid X receptor (RXR), which is activated only upon interaction with an agonistic ligand (canonical regulation) [15]. These include unsaturated fatty acids [16], prostaglandin I_2_ (prostacyclin) [17], 15-hydroxyeicosatetraenoic acid (15-HETE) [18] and a range of synthetic ligands, originally developed in light of the association of PPARβ/δ with metabolic diseases [15]. Genome-wide analyses have identified PPRE-mediated repression as a major mechanism of transcriptional regulation by unliganded PPARβ/δ, and showed that an agonist-mediated switch induces a subset of these genes [19]. PPRE-mediated repression is enhanced by inverse agonists, such as ST247 [20], which establish a repressor complex that apparently is different from the unliganded receptor complex [21].

PPARβ/δ can also regulate genes by interacting with specific transcription factors both in a PPRE-dependent [22] and independent fashion [23]. For example, unliganded PPARβ/δ in murine macrophages sequesters BCL6, a transcriptional repressor of inflammatory NFκB-regulated genes [23]. PPARβ/δ also modulates NFκB signaling by other mechanisms, including its interaction with the p65 subunit of NFκB [24-27].

We have recently addressed the function of PPARβ/δ in normal human macrophages by determining the global PPARβ/δ-regulated signaling network in primary monocyte-derived macrophages [28]. Besides canonically regulated genes with metabolic functions, we also identified a number of target genes with immune regulatory functions. These are type-selective and subject to either canonical regulation, such as *CD1D, CD52, CD300A, LRP5, NLRC,* or indirect repression by agonists, mainly affecting NFκB and STAT target genes. Consistent with these findings, PPARβ/δ agonists triggered hallmarks of an anti-inflammatory phenotype. However, we also identified positive regulatory effects on specific immune modulatory modules, in particular a stimulation of T-cell activation. PPARβ/δ agonists thus induce a unique macrophage activation state with strong anti-inflammatory but also specific stimulatory components, suggesting a context-dependent function of PPARβ/δ in immune regulation.

To date, transcriptome data for human TAMs has not been reported. Furthermore, the gene regulatory function of PPARβ/δ in TAMs has not been analyzed. Ovarian cancer is an excellent model to study TAMs, since these cells can be isolated in large quantities from the malignancy-associated peritoneal ascites. These ascites-derived macrophages display a mixed-polarization phenotype expressing both M1 and M2 markers [8]. Consistent with this finding, interpatient polarization differences unrelated to the M1/M2 classification scheme showed a clear association with the clinical outcome [8]. To elucidate the mechanisms underlying the pro-tumorigenic polarization of TAMs in ovarian cancer and the role of PPARβ/δ in this context we determined the PPARβ/δ-regulated transcriptome and PPARβ/δ cistrome in ovarian carcinoma TAMs in comparison to normal human monocyte-derived macrophages (MDMs).

## RESULTS

### Ligand-induced cellular alterations in human MDMs

**Figure 1:**
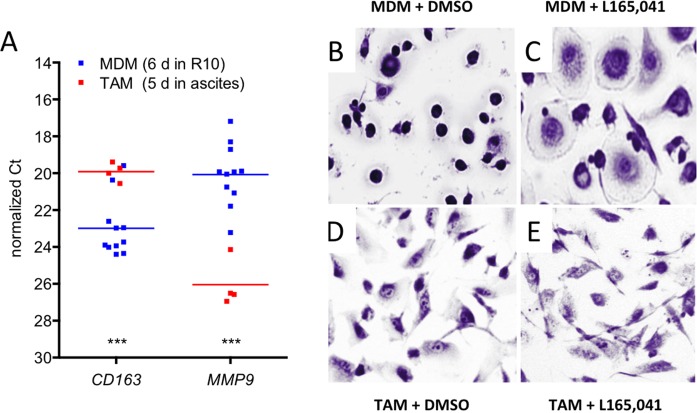
Effects of PPARβ/δ ligands on the morphology of human MDMs and ovarian carcinoma TAMs **A**. Expression of the macrophage polarization marker genes *CD163* and *MMP9* in cultured TAMs and MDMs. The data were obtained by RT-qPCR analysis of TAMs (red data points; *n* = 4) and MDMs (blue: *n* = 11) from different donors. Horizontal lines show the medians; asterisks indicate statistical significance. **B**, **C**. Giemsa staining of human MDMs differentiated in XV0 medium for 8 days in the presence of the PPARβ/δ agonist L165,041 or solvent (DMSO). **D**, **E**. TAMs treated with agonist or DMSO as in panel B and C.

CD14^+^ cells from human serous ovarian carcinoma ascites (TAMs) rapidly adhere to cell culture dishes and assume a macrophage-like morphology. We used this experimental system to investigate the affects of the synthetic PPARβ/δ agonist L165,041 on freshly isolated TAMs in short-term culture in comparison to normal monocyte-derived macrophages (MDMs). This comparison is conceptually relevant, since TAMs, including ascites-associated macrophages, are derived from blood monocytes [29-32]. Under the experimental conditions used TAMs showed a clearly enhanced expression of *CD163* and a very low level of *MMP9* mRNA relative to MDMs (Figure 1A), which is consistent with the polarization phenotype of TAMs *in vivo* [8]. We therefore conclude that our experimental system is suitable to investigate ligand-induced changes in TAMs compared to MDMs.

We have previously described that the synthetic PPARβ/δ agonist L165,041 induces a morphology in MDMs that resembles that of IL-4 treated macrophages [28] (Figure 1B and 1C). TAMs, on the other hand, displayed an unchanged morphology upon L165,041 treatment (Figure 1D and 1E). This observation suggests that TAMs are largely unresponsive to exogenous PPARβ/δ ligands. In order to address the mechanistic basis of this observation we performed comprehensive genome-wide studies as described below.

### Impaired ligand response and upregulation of PPARβ/δ target genes in cultured ovarian carcinoma TAMs

**Figure 2:**
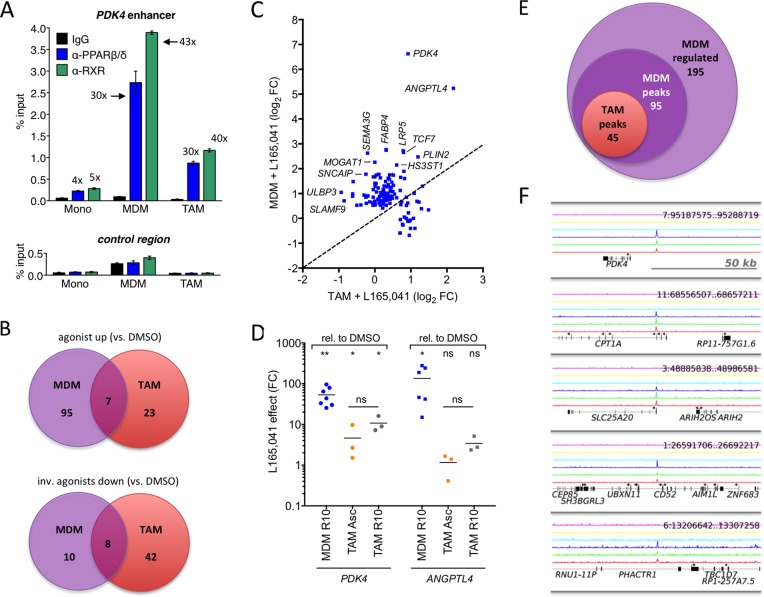
Deregulation of PPARβ/δ target genes in cultured ovarian carcinoma TAMs **A**. PPARβ/δ and RXR enrichment at the *PDK4* enhancer and an irrelevant control region in human monocytes, MDMs and TAMs (ChIP-qPCR; sample size: 4). **B**. Venn diagrams of RNA-Seq data showing overlaps of ligand-regulated high-confidence direct target genes in MDMs grown in R10 medium or purified TAMs cultured in ascites for 1 day in the presence of agonist (L165,041), inverse agonist (ST247or PT-S264) or solvent (DMSO). **C**. Ligand response of PPARβ/δ target genes in TAMs versus MDMs. Data represents the log_2_ fold change (L165,041 relative to DMS0) calculated from RNA-Seq data. The diagonal line indicates equal regulation in both cell types. **D**. Expression and ligand response of *PDK4* and *ANGPTL4* by L165,041 in MDMs in R10 (*n* = 7) and TAMs (*n* = 3) cultured in either ascites or R10 medium. Cells were cultured in the presence of ligand or DMSO for 24 h and analyzed by RT-qPCR. Data are expressed as fold regulation (FC) relative to DMSO-treated cells. **E**. Overlap of genes regulated in MDMs (agonist versus inverse agonist), genomic regions with PPARβ/δ binding sites in MDMs and PPARβ/δ enrichment sites in TAMs (ChIP-Seq). **F**. PPARβ/δ enrichment (ChIP-Seq) at the *PDK4, CPT1A, SLC25A20, CD52* and *PHACTR1* loci for 3 different TAM samples (bottom 3 lines: dark blue, green, red). The top 3 lanes (magenta, yellow, light blue) represent the corresponding control IgG runs.

Ascites-derived adherent macrophages showed a clear accumulation of PPARβ/δ and RXR at the upstream enhancer of the established PPARβ/δ target gene *PDK4* [19, 33] *in vivo* (Figure 2A) with a strong enrichment of both factors (30-fold relative to IgG control for PPARβ/δ; 40-fold for RXR). This is similar to the enrichment in MDMs (30- and 43-fold, respectively), but much higher compared to monocytes (4- to 5-fold, respectively). These data are therefore consistent with the definition of ascites-derived CD14^+^ cells as TAMs rather than ascites-associated monocytes and confirm their suitability for PPARβ/δ centered genome-wide studies.

Toward this end, MDMs in normal growth medium and freshly isolated TAMs in ascites were exposed to a synthetic PPARβ/δ agonist, inverse PPARβ agonists or solvent (DMSO) for 1 day and analyzed by RNA-Seq (Table S2). The specificity of these ligands for PPARβ/δ is illustrated in Figure S1. Only a small number of genes (*n* = 30) were found to be induced by the agonist L165,041 in TAMs (logFC≥1; FPKM≥0.3) compared to MDMs (*n* = 102) with a small intersection (*n* = 7; Figure 2B, top; Figure 2C; Table S3). On the other hand, the number of genes downregulated by the inverse agonists ST247 or PT-S264 was considerably greater in TAMs (*n* = 50) relative to MDMs (*n* = 18) with a minor overlap (*n* = 8; Figure 2B, bottom; Table S3). These findings would be consistent with the presence of high concentrations of PPARβ/δ agonists in TAMs relative to MDMs.

The observation that the majority of PPARβ/δ target genes were refractory to synthetic agonists was confirmed by RT-qPCR for *PDK4 and ANGPTL4* (Figure 2D). Both genes were induced by L165,041 in MDMs >50-fold (average; blue symbols), whereas induction in TAMs cultured in ascites (orange symbols) was <10-fold (*PDK4*) or undetectable (*ANGPTL4*). When TAMs were cultured in R10 for 24 h instead of ascites, *PDK4* induction was only slightly higher (grey symbols). These findings indicate that the loss of ligand regulation in TAMs is not dependent on the continuous presence of ascites, pointing to a relatively stable alteration affecting the regulation of PPARβ/δ target genes.

We have previously identified canonical PPARβ/δ target genes in human MDMs that are agonist-induced and occupied by PPARβ/δ-RXR complexes [28]. In combination with the additional RNA-Seq data of the present study, a total of 195 ligand-regulated target genes were identified, defined as “upregulated by agonist versus inverse agonist”, 95 of which were associated with PPARβ/δ enrichment sites (Figure 2E; Table S3, columns “L” and “K”). Delineation of the PPARβ/δ cistrome for 3 different patient samples in the present study (Suppl. Table S4) showed that at least 45 of these genomic loci were also occupied by PPARβ/δ in TAMs (Figure 2E; Table S3, column “J”), including those genes showing an altered ligand regulation in TAMs, exemplified by *PDK4, CPT1A, SLC25A20, CD52* and *PHACTR1* (Figure 2F).

### Deregulation of PPARβ/δ target genes in ovarian carcinoma TAMs *in vivo*

**Figure 3:**
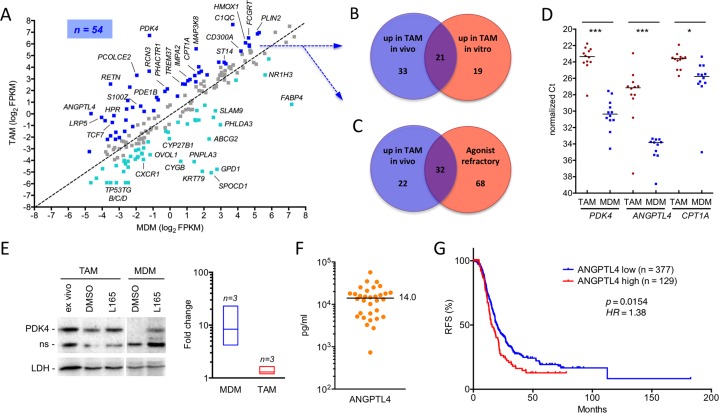
Deregulation of PPARβ/δ target genes in ovarian carcinoma TAMs *in vivo* **A**. Expression of PPARβ/δ target genes (median FPKM values) in freshly isolated TAMs (median of 10 samples) versus MDMs (5 samples). The diagonal line indicates equal levels in both cell types. Blue dots: upregulation in TAMs ≥2-fold; cyan dots: downregulation ≥2-fold in TAMs; grey dots: no change. **B**. Overlap of PPARβ/δ target genes upregulated in freshly isolated TAMs versus MDMs (blue dots in A) and in cultured TAMs (experimental setup as in Figure 2). **C**. Overlap of PPARβ/δ target genes upregulated in TAMs versus MDMs (blue dots in A) and target genes refractory to synthetic agonists in TAMs (data from Figure 2B). **D**. RT-qPCR analysis of *PDK4, ANGPTL4* and *CPT1A* mRNA expression levels in freshly isolated TAMs and MDMs from ovarian cancer patients (*n* = 12) and healthy donors (*n* = 12), respectively. Horizontal bars indicate the median. Statistical significance was tested between the respective TAM and MDM groups. **E**. Immunoblot analysis of PDK4 protein induction by PPARβ/δ agonist in MDMs and TAMs. The figure shows representative immunoblots (including PPARβ/δ and LDH as the loading control) for both cell types and a quantitative evaluation of biological replicates with TAMs from 3 different patients and MDMs from 3 donors. Cells were exposed to ligands for 1 d in R10 medium; TAMs were also analyzed directly after isolation (“*ex vivo*”). Signal intensities were quantified and standardized to LDH. The diagram on the right depicts the induction by L165,041 (fold change) in TAMs and MDMs *in vitro*; boxes show the ranges of inducibility and the median for each group of samples. Induction values for MDMs represent estimations due to the extremely low basal level of PDK4 in MDMs. The α-PDK4 antibody was validated as shown in Figure S2. n.s., non-specific band. **F**. Concentrations of ANGPTL4 protein in the ascites of serous ovarian carcinoma patients (*n* = 32) determined by ELISA. The horizontal line indicated the median. **G**. Meier-Kaplan plot showing a correlation of high *ANGPTL4* expression with the relapse-free survival of high grade serous ovarian carcinoma patients of the TCGA cohort (*n* = 377 in *ANGPTL4* high group; *n* = 129 *ANGPTL4* low) [62].

We next compared the expression and ligand regulation of PPARβ/δ target genes in ascites-associated macrophages from ten different patients (Table S5) with the set of 195 ligand-regulated target genes in MDMs identified by RNA-Seq analysis of cells from 5 healthy donors (see above; Table S3). Intriguingly, a large fraction of these PPARβ/δ target genes (dark blue dots; *n* = 54) were upregulated (log_2_FC ≥0.7) in freshly isolated TAMs relative to MDMs (Figure 3A). Approximately half of the genes upregulated in cultured TAMs (21/40) overlapped with the genes upregulated *in vivo* (Figure 3B; Table S3), thus validating the results obtained *in vitro*. Most of the genes upregulated in TAMs were also refractory to regulation by a synthetic agonist (*n* = 32; Figure 3C; Table S3), suggesting a link between upregulation and loss of ligand regulation. A summary of these data is shown in Table 1.

**Table 1:** PPARβ/δ target genes upregulated^1^ in ovarian cancer TAMs

Gene	Description	agonist MDM (FC)^2^	PPARβ/δ peak^3^3	refractory in TAM^4^
*ACADVL*	acyl-CoA dehydrogenase, very long chain	3.3	+	+
*ACSS3*	acyl-CoA synthetase short-chain family member 3	2.3	−	+
*AMOTL1*	angiomotin like 1	1.9	−	+
*ANGPTL4*	angiopoietin-like 4	37.8	+	+
*ANKRD1*	ankyrin repeat domain 1 (cardiac muscle)	1.8	−	−
*C19orf59*	chromosome 19 open reading frame 59	6.7	+	+
*C1orf162*	chromosome 1 open reading frame 162	2.2	+	+
*C1QC*	complement component 1, q subcomponent, C chain	1.5	−	−
*CABLES1*	Cdk5 and Abl enzyme substrate 1	3.2	−	+
*CACNB1*	calcium channel, voltage-dependent, beta 1 subunit	2.4	+	+
*CD300A*	CD300a molecule	1.5	+	−
*CLDND2*	claudin domain containing 2	2.2	+	+
*CPT1A*	carnitine palmitoyltransferase 1A (liver)	3.4	+	+
*CXorf21*	chromosome X open reading frame 21	1.8	+	+
*DLG4*	discs, large homolog 4 (Drosophila)	1.6	+	+
*FAM3B*	family with sequence similarity 3, member B	2.7	−	+
*FCGR3A*	Fc fragment of IgG, low affinity IIIa, receptor (CD16a)	1.5	+	−
*FCGRT*	Fc fragment of IgG, receptor, transporter, alpha	1.5	+	−
*FOS*	FBJ murine osteosarcoma viral oncogene homolog	1.1	+	−
*GPA33*	glycoprotein A33 (transmembrane)	1.8	−	+
*HMOX1*	heme oxygenase (decycling) 1	1.3	+	−
*HP*	haptoglobin	2.2	−	−
*HPR*	haptoglobin-related protein	2.6	−	−
*HS3ST1*	heparan sulfate (glucosamine) 3-O-sulfotransferase 1	4.4	−	+
*IL27*	interleukin 27	1.2	−	−
*IMPA2*	inositol(myo)-1(or 4)-monophosphatase 2	2.6	+	+
*INF2*	inverted formin, FH2 and WH2 domain containing	1.5	−	+
*KBTBD11*	kelch repeat and BTB (POZ) domain containing 11	1.3	−	−
*KLF11*	Kruppel-like factor 11	1.4	−	−
*KRT4*	keratin 4	1.9	−	+
*LRP5*	low density lipoprotein receptor-related protein 5	6.6	+	+
*MACC1*	metastasis associated in colon cancer 1	1.8	+	−
*MAP3K8*	mitogen-activated protein kinase kinase kinase 8	1.5	−	+
*MEGF9*	multiple EGF-like-domains 9	1.5	+	−
*MS4A14*	membrane-spanning 4-domains, subfam. A, member 14	1.6	−	+
*MS4A7*	membrane-spanning 4-domains, subfamily A, member 7	1.6	−	−
*PCOLCE2*	procollagen C-endopeptidase enhancer 2	1.9	−	−
*PDE1B*	phosphodiesterase 1B, calmodulin-dependent	2.2	−	−
*PDK4*	pyruvate dehydrogenase kinase 4	99.0	+	+
*PHACTR1*	phosphatase and actin regulator 1	3.1	+	+
*PLIN2*	perilipin 2	5.5	+	+
*PPP1R15B*	protein phosphatase 1, regulatory subunit 15B	1.6	+	−
*RBP7*	retinol binding protein 7, cellular	1.8	−	+
*RCN3*	reticulocalbin 3, EF-hand calcium binding domain	2.6	+	+
*RETN*	resistin	1.3	+	+
*S100Z*	S100 calcium binding protein Z	3.1	+	−
*SIPA1L2*	signal-induced proliferation-associated 1 like 2	2.1	+	+
*ST14*	suppression of tumorigenicity 14 (colon carcinoma)	2.4	+	+
*TCF7*	transcription factor 7 (T-cell specific, HMG-box)	6.3	+	+
*TMEM150B*	transmembrane protein 150B	1.2	+	−
*TMEM37*	transmembrane protein 37	1.7	+	+
*TRIM14*	tripartite motif containing 14	1.6	−	+
*TSKS*	testis-specific serine kinase substrate	0.8	+	−
*VSIG10L*	V-set and immunoglobulin domain containing 10 like	1.4	+	−

**Figure 4:**
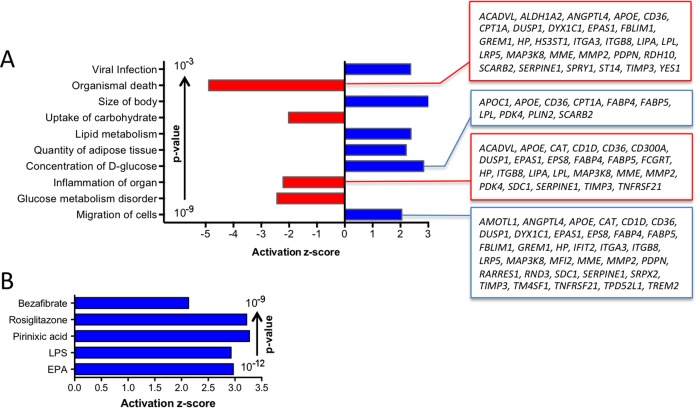
Pathway analyses of PPARβ/δ target genes constitutively upregulated in TAMs **A**. IPA *Diseases and Functions Annotation* (functionally different clusters with lowest p-values and highest z-scores). Gene names are shown for the clusters with the largest number of genes. **B**. IPA *Upstream Regulator Analysis* (5 top regulators by p-value; z-score >2).

1 LogFC TAMs in vivo vs MDMs > 0.7 (Figures 4A and 4B; Tables S3, S5)

2 Ratio FPKM L165,041 / FPKM DMSO in MDMs (Figure 2B; Table S2)

3 Peak in MDMs or TAMs: ChIP-Seq data (Figures 2E ad 2F; Table S4; Adhikary et al., 2015)

4 Refractory to synthetic agonist in TAMs (Figure 3C; Table S3); <2.0-fold (Fig. 2D, 4A, 4C; Table S2).

Comparison of the expression levels of three PPARβ/δ target genes, *PDK4, ANGPTL4* and *CPT1A* in TAMs from 12 patients and MDMs from 12 healthy donors confirmed this result (Figure 3D). As shown for *PDK4*, deregulation of gene expression in TAMs correlated with increased protein levels, which, in contrast to MDMs, were largely insensitive to ligand stimulation (Figure 3E).

Interestingly, we also found a number of PPARβ/δ target genes downregulated in TAMs relative to MDMs, for example *FABP4* and *ABCG2* (Figure 3A; cyan data points). Ovarian cancer is known to consist of a plethora of signaling mediators, including cytokines [8] and lipids (see data below). It is therefore likely that a subset of target genes is downregulated by repressive signaling pathways triggered by specific components of the ovarian cancer microenvironment, thereby preventing their potential stimulation analogous to the PPARβ/δ target genes discussed in the preceding paragraph.

The deregulation of *ANGPTL4* is of particular interest, since its secreted product has been associated with cancer cell invasion and metastasis and is present in substantial amounts in the malignancy-associated ascites of most serous ovarian carcinoma patients (Figure 3F). We therefore tested the Cancer Genome Atlas (TCGA) cohort of 506 high grade serous ovarian cancer patients [34] for a potential link of *ANGPTL4* expression to the clinical outcome of the disease. As depicted by the Kaplan-Meier plot in Figure 3G, *ANGPTL4* levels showed a significant inverse association with relapse-free survival (RFS) [p = 0.0154; hazard ratio = 1.38 (1.06-1.79); median RFS: 15.63 versus 19.8 months].

Annotation of all PPARβ/δ target genes constitutively upregulated in TAMs by Ingenuity Pathway Analysis (IPA) identified metabolism (glucose, lipid), inflammation, cell migration and survival as top functions (Figure 4A). As expected, the PPAR ligands (benzafibrate, EPA, rosiglitazone, pirinixic acid) were found among the top upstream regulators (Figure 4B). The presence of the pro-inflammatory mediator LPS in this list is consistent with the results obtained by the functional annotation analysis (inflammation).

### Deregulation of PPARβ/δ target genes by soluble mediators in malignancy-associated ascites

**Figure 5:**
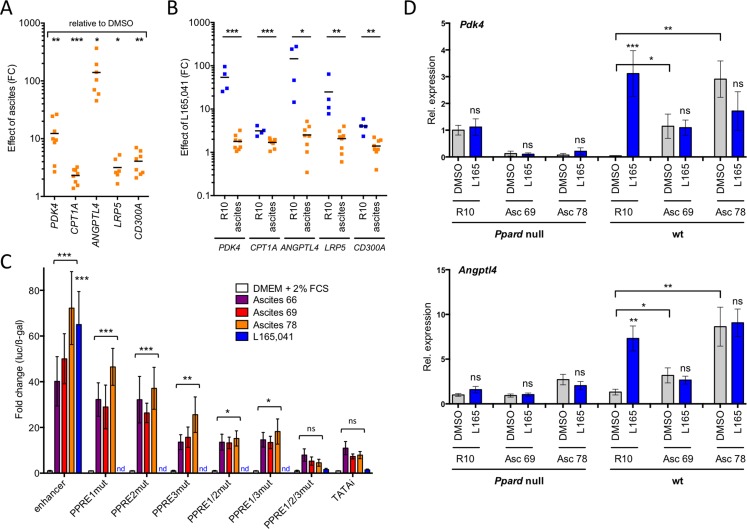
Ascites deregulates PPARβ/δ target genes in normal macrophages and in a PPARβ/δ-dependent fashion **A**. Upregulation of PPARβ/δ target genes by ascites in MDMs (*n* = 8; 4 different MDM samples; 2 different ascites samples). RT-qPCR data are expressed as fold change (FC) relative to MDMs R10 medium. **B**. Regulation of target genes by L165,041 in MDMs (*n* = 4) in R10 or ascites (2 different samples). Data indicate FC relative to DMSO-treated cells. **C**. PPRE-dependent induction of a *PDK4* enhancer-luciferase construct in transiently transfected HEY cells (*n* = 3). Constructs were mutated in either 1, 2 or all 3 PPREs, as indicated. Data were normalized to β-galactosidase activity from a co-transfected CMV-β-gal expression vector. **D**. Response of the direct PPARβ/δ target genes *Pdk4* and *Angptl4* to two different ascites samples and L165,041 in bone marrow-derived macrophages from wild-type and *Ppard* null mice (sample size: 3 each). Statistical significance was tested for induction by ascites relative to DMSO-treated cells in **C** and **D** (asterisks/ns above square brackets) and for induction by L165,041 or in D (asterisks/ns above blue bars).

The data in Figure 2 suggests that the unaltered occupancy of direct target genes by PPARβ/δ-RXR in conjunction with a TAM-specific mechanism activating these chromatin-bound complexes is responsible for their deregulation in TAMs. One explanation for this deregulation could be the presence of ascites-associated activators of PPARβ/δ. We addressed this question by testing the effect of cell-free ascites samples on the regulation of PPARβ/δ target genes in MDMs. Figure 5A shows a clear upregulation of the target genes *PDK4, CPT1A, ANGPTL4, LRP5 and CD300A* by two different ascites samples, which in several cases reached the level of L165,041 induction (Figure 5B; blue dots). Furthermore, induction of all 5 genes by L165,041 was severely diminished in the presence of ascites (Figure 5B; orange dots).

Therefore, we sought to investigate whether deregulation of target genes by ascites might be attributable to the activation of PPARβ/δ, and thus dependent on PPARβ/δ binding sites (PPREs) in these genes. It has previously been shown that an upstream enhancer with three contiguous PPREs mediates induction of *PDK4* by PPARβ/δ ligands [19]. A luciferase construct with a genomic 1.5 kb fragment encompassing this enhancer showed a dramatic upregulation by three different ascites samples (Figure 5C). These effects were clearly PPRE-dependent, since the mutation of 1, 2 or 3 sites gradually abrogated the induction of luciferase activity by ascites (Figure 5C).

We found that PPARβ/δ target genes are inducible by ascites in murine bone marrow-derived macrophages (BMDMs), similar to human MDMs. We were therefore able to show that the observed target gene deregulation was dependent on functional PPARβ/δ. Ascites upregulated the *Pdk4* and *Angptl4* genes and abrogated their induction by L165,041 in wild-type BMDMs, whereas no significant ascites effect was detected on *PDK4* in cells with disrupted *Ppard* alleles (Figure 5D). Likewise, the ascites-mediated induction of *ANGPTL4* was either absent (Asc69) or strongly reduced (Asc78) in *Ppard* null cells. These observations indicate that PPARβ/δ is responsible for the deregulation of PPARβ/δ target genes by ascites, even though a minor contribution by other PPAR subtypes cannot be unequivocally ruled out. *ANGPTL4* is induced by a plethora of signaling pathways [35], which presumably explains the residual induction by Asc78 in Ppard null cells.

### Endogenous agonists present in ovarian carcinoma ascites deregulate PPARβ/δ target genes in MDMs

**Figure 6:**
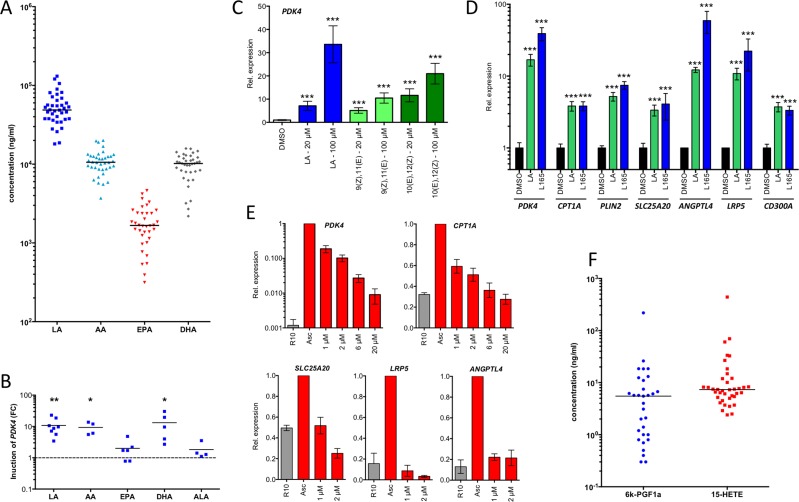
PPARβ/δ ligands are present in ascites at high concentrations and induce PPARβ/δ target genes **A**. LC-MS/MS analysis of polyunsaturated fatty acids (PUFAs) in ascites from ovarian carcinoma patients (*n* = 38). **B**. Induction of *PDK4* in MDMs after 24 h exposure to different PUFAs in different donors (*n* = 4-8). Each data point represents a biological replicate. **C**. Rapid induction (3 h stimulus) of *PDK4* by LA and conjugated 9(Z),11(E)-LA and 10(Z),12(E)-LA in MDMs (triplicates). **D**. Induction of PPARβ/δ target genes in MDMs after 24 h exposure to linoleic acid (LA) in comparison to L165,041 (triplicates). **E**. Repression of PPARβ/δ target genes in MDMs (*n* = 3) cultured in ascites for 48 h by different concentrations of PT-S264 added during for the last 24 h of the experiment. Values were normalized to 1 for cells in ascites. **F**. LC-MS analysis of 15-HETE and the stable prostacyclin derivative 6k-PGF1α in the same samples as in A. Horizontal bars show the medians in panels A and B. Values represent averages of triplicate measurements ± standard deviation in all panels. Significance was tested relative to control cells.

The results described above suggest that ovarian cancer associated ascites might contain high levels of endogenous PPARβ/δ agonists. Since all known PPARβ/δ agonists are fatty acids or fatty acid derivatives, we performed a systematic lipidomic analysis of 97 molecules in 38 different ascites samples by LC-MS/MS (Suppl. Table S6). This analysis revealed consistently very high concentrations of several polyunsaturated fatty acids (PUFAs) known as PPARβ/δ agonists [16], with the highest levels observed with linoleic acid (LA) (Figure 6A). The median concentration for LA was ~50 μg/ml (~180 μM), which is far above the described IC_50_ of 0.75 μM for PPARβ/δ binding [16]. This also applies to arachidonic acid (AA) and docosahexaenoic acid (DHA) with median ascites concentrations around 10 μg/ml (Figure 6A).

Addition of AA, LA or DHA to MDM cultures at a concentration of 20 μM for 24 h resulted in a strong induction of the *PDK4* gene, while eicosapentaenoic acid (EPA) and α-linolenic acid (ALA) had only very modest effects (Figure 6B). *PDK4* induction by LA was dose-dependent and rapid with a nearly 10-fold induction already after 3 h (Figure 6C). Similar results were obtained with the conjugated LAs 9(Z),11(E)LA and 10(Z),12(E)LA (Figure 6C). LA also potently induced other direct PPARβ/δ target genes, and this induction was close, or even equal, to activation by L165,041, as shown for *PDK4, CPT1A, PLIN2, SLC25A20, ANGPTL4, LRP5 and CD300A* in Figure 6D.

A number of PPARβ/δ target genes deregulated by ovarian cancer ascites have functions in oncogenesis and immune regulation. It was therefore of great interest to investigate whether their overexpression could be reverted by inverse PPARβ/δ agonists in spite of the high concentrations of agonists in ascites. As shown in Figure 6E, treatment of MDMs cultured in ascites with increasing concentrations of PT-S264 for 24 h led to progressively lower levels of *PDK4* mRNA expression. At the highest tested concentration (20 μM), expression was reduced to less than 5%. Likewise, *CPT1A, SLC25A20, LRP5* and *ANGPTL4* mRNA expression was reduced to basal levels by PT-S264, with *LRP5* and *ANGPTL4* being strongly repressed already at concentrations of 1 μM. These results clearly indicate that inverse agonists are suitable to counteract the deregulation of PPARβ/δ target genes in ovarian carcinoma TAMs.

We also found two other endogenous PPARβ/δ agonists, 15-HETE [18] and 6-keto-prostglandin F_1α_ (6-kPGF_1α_), the stable degradation product of prostacyclin [17, 36] in all ascites samples (Figure 6F). Both, 6-kPGF_1α_ and 15-HETE were found at median levels of ~10 ng/ml (~30 nM), which corresponds to approximately 3% of the IC_50_ concentrations required for PPARβ/δ activation [18, 36]. Both metabolites are therefore unlikely to play a role in the deregulation of PPARβ/δ target genes in TAMs.

### Fatty acid accumulation in lipid droplets correlates with transcriptional deregulation

**Figure 7:**
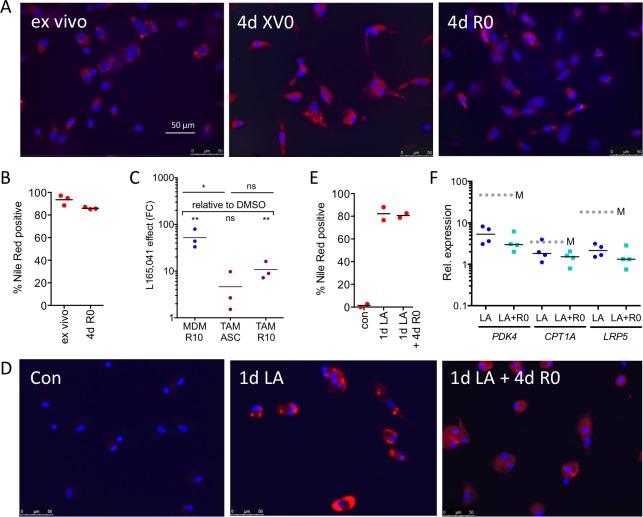
Association of the stable accumulation of lipid droplets in TAMs with the deregulation of the PPARβ/δ target gene PDK4 **A**. Staining of primary TAMs with Nile Red 0 h (*ex vivo*) and 4 d after plating in serum-free XV0 or R0 medium. **B**. Quantification of Nile Red stained TAMs (*n* = 3) treated as in **A**. **C**. L165,041 induction of *PDK4* in MDMs (*n* = 3) and in TAMs (*n* = 3) cultured for 4d in ascites or R10 medium. **D**. Staining of MDMs with Nile Red before (d0) and after a 24-hour exposure to LA (d1), followed by a 4d fatty acid withdrawal in serum-free R0 medium (d1+4). **E**. Quantification of Nile Red stained MDMs (*n* = 2) before and after LA exposure as in **D**. **F**. L165,041 induction of PPARβ/δ target genes in MDMs (*n* = 4) pretreated with LA for 1 d, followed by a 4d serum-free R0 medium lacking fatty acids.

The data in Figure 2D showed that ligand regulation in TAMs can only be partially restored by culturing the cells in normal cell culture medium. Since macrophages have a propensity to accumulate intracellular lipids, which is enhanced by PPARβ/δ [37], we tested this for ovarian carcinoma TAMs. As shown by staining with the fluorescent dye Nile Red, ascites-derived TAMs harbor a huge amount of lipid droplets, which remains basically unchanged upon culturing these cells in normal growth medium for 4 days (Figure 7A, 7B). The stability of lipid droplets correlated with a compromised ligand regulation of the PPARβ/δ target gene *PDK4* (Figure 7C). Consistent with this finding, MDMs rapidly accumulate lipid droplets when exposed to LA at a high level found in ascites, which persisted upon withdrawal of the fatty acids (Figure 7D, 7E), concomitantly with an impaired inducibility by synthetic ligands (Figure 7F). It is therefore likely that internalization of PUFAs from the tumor microenvironment generates a reservoir of agonists contributing to a stable upregulation of PPARβ/δ target genes.

## DISCUSSION

PPARβ/δ regulates a large group of genes with functions in intermediary metabolism, inflammation and tumor progression, which are coordinately upregulated in TAMs by PUFA ligands present at high concentrations in the ascites of ovarian cancer patients (Table 1). Functional annotation analyses showed that these genes are not only associated with cell type-independent roles in energy production, fatty acid oxidation and lipid storage, but also figure in inflammation, cell migration and cell survival. Upregulation of several of these genes in TAMs is compatible with the pro-tumorigenic role of TAMs and may serve not only to skew TAM polarization but may also directly promote tumor progression, for instance via the secretion of soluble mediators, such as ANGPTL4. We therefore propose that the deregulation of PPARβ/δ target genes by mediators of the tumor environment acts in conjunction with other signaling mechanisms to effect the pro-tumorigenic conversion of host-derived monocytic cells.

### Fatty acid PPARβ/δ ligands in ascites

Several PUFAs known to act as PPARβ/δ agonists were found in all ascites samples tested at levels exceeding the concentrations required for maximal PPARβ/δ activation, in particular LA, but also arachidonic acid and docosahexaenoic acid [16]. High levels of lipoprotein complexes in ovarian cancer ascites have been described in a previous study, but their fatty acid composition was not determined [38]. Another report suggests the mobilization of LA from omentum in ovarian cancer patients [39], consistent with the very high levels of this fatty acid in the malignancy-associated ascites found in the present study. Several studies also indicate that fatty acids are relevant to the biology and clinical outcome of ovarian cancer. Thus, the increased expression of the fatty acid synthase gene (*FAS*) predicts shorter survival [40], dietary fat intake and altered lipid metabolism are linked to ovarian cancer risk [41] and in a mouse model tumor growth and invasion are fueled by direct transfer of lipids from omental adipocytes to ovarian cancer with a key role for fatty acid-binding protein 4 [42].

Blood plasma also contains high concentrations of PUFAs [43], yet PPARβ/δ target genes are expressed at low levels in blood monocytes, which is presumably due to the low level of PPARβ/δ expression in monocytes (Figure 2A and [28]), at least in part. TAMs represent a special situation in that these cells express PPARβ/δ at readily detectable levels and at the same time are exposed to high levels of ligands in the tumor microenvironment. Our findings also suggest that PPARβ/δ may serve as a marker to distinguish monocytes from macrophages, and also support the conclusion that ascites-associated CD14^+^ cells are macrophages rather than monocytes.

### Deregulated PPARβ/δ target genes in TAMs

The target gene *ANGPTL4* [44, 45] is of particular interest in the context of the present study, since it not only figures in lipid metabolism as a regulator of lipoprotein lipase, but also plays an apparently essential role in tumor progression [46]. Thus, ANGPTL4 secreted by tumor cells in response to TGF-β and released into the circulation increases the permeability of lung capillaries and facilitates the extravasation of disseminated breast cancer cells in a mouse model [35]. Furthermore, *ANGPTL4* increases cancer cell invasion [21] and is part of gene expression signatures associated with distant metastasis in human cancer patients [35, 47]. ANGPTL4 also inhibits anoikis, which is essential for the survival of circulating tumor cells [48]. Consistent with these observations, several oncogenic signaling pathways converge on the *ANGPTL4* gene, including TGFβ [21, 35, 45] and AP1 [45].

Deregulated PPARβ/δ target genes with potential roles in macrophage regulation are *CD300A* and *FOS*. CD300A is a membrane glycoprotein with anti-inflammatory functions. For example, deletion of the *Cd300a* gene in mice has been shown to result in pro-inflammatory activation of peritoneal macrophages [49], suggesting that its upregulation in TAMs has an immune suppressive effect. On the other hand, FOS has been strongly associated with the pro-inflammatory activation of macrophages [50]. These observations are compatible with a role of deregulated PPARβ/δ target genes in mediating the mixed-polarization phenotype of TAMs [4, 5, 7, 8].

Several other novel PPARβ/δ target genes upregulated in TAMs potentially play a role in promoting macrophage migration. i.e., *PHACTR1* (phosphatase and actin regulator 1), *MACC1* and *ST14*. PHACTR1 plays a role in the G-actin mediated control of actomyosin assembly [51], MACC1 is a transcriptional activator of *MET* (HGF receptor) and acts as a key regulator of cell motility [52], and ST14/epithin is a protease transcriptionally induced in macrophages by pro-inflammatory pathways to mediate transendothelial migration [53].

Another PPARβ/δ target gene upregulated in TAMs is *LRP5*. Its product LRP5 acts as a Frizzled co-receptor and activator of Wnt signaling [54]. In macrophages, LRP5 is involved in the innate inflammatory reaction to lipid infiltration by activating the Wnt pathway and promoting lipid uptake, leading to the formation of foam cells [55]. It is possible that the deregulation of LRP5 in TAMs contributes to the intracellular accumulation of fatty acids in lipid droplets observed in the present study.

Finally, the dramatic upregulation of *PDK4* probably affect energy metabolism in TAMs such that glucose catabolism is shifted towards glycolysis and lactate production (Warburg effect) [56]. This would render TAMs largely independent from the availability of oxygen, thus endowing the cells with the ability to cope with the hypoxic conditions frequently encountered in the tumor microenvironment.

Our data also show that a large number of target genes that are deregulated by ovarian cancer ascites are repressed by inverse PPARβ/δ agonists, with PT-S264 being able to suppress these genes below the basal level observed in the absence of ascites. Since several of these genes have functions in disease-associated processes as discussed above, inverse PPARβ/δ agonists may represent invaluable experimental tools to interfere with the tumor-promoting effects of the ovarian cancer microenvironment.

### Expression of indirect PPARβ/δ target genes in TAMs

A large group of PPARβ/δ target genes in macrophages is repressed by PPARβ/δ agonists independent of direct DNA contacts (see Introduction). These genes are mostly associated with pro-inflammatory functions exerted by macrophages. In TAMs these inverse target genes are also frequently deregulated and refractory to synthetic ligands. However, the underlying mechanisms are complex, as indicated by the extreme variability of expression levels, ligand inducibility and ascites effects observed for different genes as well as individual patients (see Suppl. Figures S3 and S4 for examples). It is likely that the inverse target genes are highly prone to such variations, since they are regulated by multiple signaling pathways that are triggered by numerous cytokines whose concentrations are highly divergent among patients. It is obvious that these variabilities contribute to the observed heterogeneity, in many cases presumably without a significant contribution of PPARβ/δ itself. To understand the mechanistic basis of the altered transcriptome of inverse PPARβ/δ target genes in TAMs it will be necessary to perform in-depth analyses of transcription factor occupancy and epigenetic modifications at individual genes and relate this data to specific pathways and mediators.

## MATERIALS AND METHODS

### Ligands

L165,041 was purchased from Biozol. ST247 was synthesized as described [20, 57]. The inverse PPARβ/δ agonist PT-S264 is an optimized derivative of ST247 with improved plasma stability (Toth et al., manuscript submitted). Synthetic ligands were used at a concentration of 1 μM in all experiments unless indicated otherwise. Cells were treated for 24 h unless indicated otherwise. PUFAs were obtained from Biomol.

### Mice

*Ppard* null and wt mice were generated by crossing floxed *Ppard* mice [58] and Sox2-Cre mice [59] as described [60]. Sox2-Cre mice were obtained from Jackson Laboratory (Bar Harbor, Maine), the floxed *Ppard* mouse strain was kindly provided by Dr. R. Evans (Salk Institute, La Jolla, CA). For genotyping the following primers were used: *Ppard* intron 3 (forward: GGC TGG GTC ACA AGA GCT ATT GTC TC), *Ppard* exon 4 (forward: GGC GTG GGG ATT TGC CTG CTT CA); *Ppard* intron 4 (reverse: GAG CCG CCT CTC GCC ATC CTT TCA G; fragment sizes: *Ppard* wt: 360 bp; *Ppard* floxed: 400 bp; *Ppard* ko: 240 bp; *Cre* (forward: CCT GGA AAA TGC TTC TGT CCG; reverse: CAG GGT GTT ATA AGC AAT CCC); fragment size: 390 bp.

### Patient samples

Peripheral blood mononuclear cells (PBMCs) were obtained from healthy adult volunteers. Ascites was collected from untreated high-grade serous ovarian carcinoma patients undergoing surgery at the University Hospital Marburg. Informed consent was obtained from all patients according to the protocols approved by the institutional ethics committee.

### Isolation of CD14^+^ cells

Mononuclear cells were isolated from ascites and peripheral blood by Lymphocyte Separation Medium 1077 (PromoCell) density gradient centrifugation and purified by magnetic cell sorting (MACS) using CD14 microbeads (Miltenyi Biotech) or adherence selection on cell culture dishes for 30 min. For ChIP experiments, TAMs were purified by adherence selection. The purity of CD14^+^ cells was > 90%. Purified TAMs and MDMs were analyzed by FACS, lysed in PeqGold (Peqlab) for RNA preparation or cultured as described below.

### Cell culture and cytokine treatment of TAMs and MDMs

CD14^+^ monocytes and TAMs were cultured either in RPMI1640 with 10% fetal bovine serum (FCS; R10 medium), serum-free RPMI1640 (R0 medium) or in serum-free macrophage X-VIVO 10 medium (XV0 medium) (Biozym Scientific). Monocyte-derived macrophages (MDMs) were differentiated from CD14^+^ monocytes of healthy volunteers for 5-7 d at 1×10^6^ cells/ml. HEY ovarian cancer cells (ATCC) were maintained in DMEM plus 10% FCS.

### Lipidomic analysis

Ascites samples (1 ml) were spiked with 100 μl deuterated internal standard and extracted using solid reverse phase extraction columns (Strata-X 33, Phenomenex). Fatty acids derivatives were eluted into 1.0 ml of methanol, lyophilized and resuspended in 100 ml of water/acetonitrile/formic acid (70:30:0.02, v/v/v; solvent A) and analyzed by LC-MS/MS on an Agilent 1290 separation system. Samples were separated on a Synergi reverse-phase C18 column (2.1×250 mm; Phenomenex) using a gradient as follows: flow rate =0.3 μl/min, 1 min (acetonitrile/isopropyl alcohol, 50:50, v/v; solvent B), 3 min (25% solvent B), 11 min (45% solvent B), 13 min (60% solvent B), 18 min (75% solvent B), 18.5 min (90% solvent B), 20 min (90% solvent B), 21 min (0% solvent). The separation system was coupled to an electrospray interface of a QTrap 5500 mass spectrometer (AB Sciex). Compounds were detected in scheduled multiple reaction monitoring mode. For quantification a 12-point calibration curve for each analyte was used. Data analysis was performed using Analyst (v1.6.1) and MultiQuant (v2.1.1) (AB Sciex).

### Immunoblotting

Immunoblots were performed following standard protocols using the following antibodies: α-PPARβ/δ (sc-74517; Santa Cruz, Heidelberg, Germany), α-PDK4 (ab110336; Abcam, Cambridge, United Kingdom), α-LDH (sc-33781; Santa Cruz, Heidelberg, Germany), α-rabbit IgG HRP-linked AB and α-mouse IgG HRP-linked AB (cs7074, cs7076; Cell Signaling, NEB, Frankfurt, Germany). ChemiDoc MP system and Image Lab software version 5 (Bio-Rad, München, Germany) were used for detection and quantification.

### Quantification of secreted ANGPTL4 protein

ANGPTL4 levels in ascites from ovarian cancer patients were determined by ELISA (Aviscera Bioscience, Santa Clara, CA), according to the instructions of the manufacturer. The antibody used in this kit recognizes the bioactive C-terminal processing product (cANGPTL4).

### Nile Red staining

Cells were stained for 10 minutes at 37 °C with 500 nM Nile Red (Biomol, Hamburg, Germany) in PBS and visualized using a Leica DM5000 B microscope. Nuclei were stained using Vecta Shield with DAPI (Biozol, Eching, Germany). For quantification the percentage of Nile Red positive cells was determined by counting 20 faces per donor or patient per treatment.

### Luciferase reporter assay

The *PDK4* upstream enhancer region was cloned into pGL3-TATAi [61] via *Kpn*I sites using the following primers:

5′-AAAGGTACCAAATGCTGAGTTTGGGCAAC and 5′-AAAGGTACCAGCCTTGTGAGCAACCAAAG. PPREs were mutated with the following primers 5′-CAGGCTAAGTTGGTGTATGGTCAGTCCCACACC, 5′-GAAGTTTAGTAGGTGTACGGTCACTGCTGCCGA and5′-AGAGCTCACTAGGGGTATGGTCGGGGAGACCAAG, and their respective reverse complement primers. HEY1 cells were transfected with the indicated reporter vector and pEF6/V5-His-TOPO/*lacZ* (Life Technologies) as described [18] and incubated overnight in DMEM with 2% FCS. On the next day, cells were washed with PBS and received either fresh medium with or without 1 μM L165,041 or ascites for 24 h. Lysates were prepared and measured according to the manufacturer's instructions (Beetle Juice Big and β-Gal Juice PLUS Kit for normalization; pjk GmbH) with an Orion L luminometer (Berthold).

### RT-qPCR and RNA-Seq

cDNA isolation and qPCR analyses were performed as described [20]. L27 was used for normalization. Primer sequences are listed in Suppl. Table S1. RNA-Seq was carried out as described elsewhere [28]. Sequencing data were deposited at EBI ArrayExpress (accession number TAM data: E-MTAB-3167; MDM data: E-MTAB-3114 and E-MTAB-3398). Data were quantile normalized using all RNA-Seq datasets. Gene model data were retrieved from Ensembl revision 74.

### Bioinformatic analysis of RNA-Seq data

We sequenced 10 TAMs samples from 10 patients directly after harvesting (“*in vivo*”), one additional TAM sample was used for ligand response experiments in autologous ascites (“*in vitro*”; L165,041, ST247 and DMSO). In addition to previously described MDM ligand response experiments from two donors [28] in R10 and X0 medium (L165,041, ST247, PT-S264, DMSO), we performed three additional sets from three donors in R10 (L165,041, PT-S264 and DMSO control). ST247 was used at a concentrations of 300 nM, all others at 1 μM.

Genes were considered for differential expression analyses only if they had an FPKM of at least 0.3 and a minimum of 50 tags in at least one sample. LogFC values for ligand experiments were calculated pairwise for individual donors. For ligand regulation in MDMs (Figure 2B) a logFC of at least 0.7 in 4 out of 5 replicates was required. Figure 2C shows median pairwise logFC data. Regulated target genes in MDMs (*n* = 195; Figures 2E and 3A) were defined as genes showing regulation in at least one of the following comparisons: agonist *vs* DMSO control (up regulated), inverse agonist *vs* DMSO control (down regulated) or agonist *vs* inverse agonist (up regulated). Figure 3A shows median FPKM values of 10 TAM samples and 5 MDM DMSO control samples. In Figure 3B and 3C, “up in TAM *in vivo*” is a subset of the canonical target genes that showed a 2-fold (1 logFC unit) difference between TAMs and MDMs. Table S5 was filtered based on t-tests between 10 TAM *in vitro* samples and 5 MDM DMSO samples (FDR/Benjamini-Hochberg ≤0.05). The set “up in TAM *in vitro*” is similarly defined as canonical target genes that (i) were upregulated (0.7 logFC) in TAM/DMSO compared to the two previously reported MDM/DMSO samples, and (ii) showed an at least 0.5 units higher FPKM value in the TAM sample compared to both MDM samples. Agonist refractory genes (Figure 3C) are agonist inducible genes in MDMs that showed no such regulation (same logFC threshold) or less than 50% induction (fold change) by L165,041 in TAMs relative to MDMs.

### ChIP-PCR and ChIP-Seq

ChIP was performed and evaluated as described using the following antibodies: IgG pool, I5006 (Sigma Aldrich); α-PPARβ/δ, sc-7197; α-RXR, sc-774 (Santa Cruz, Heidelberg, Germany). ChIP-Seq, mapping of ChIP-Seq reads and peak calling were carried out as described [28].

### Bioinformatic analysis of ChIP-Seq data

ChIP-Seq peaks were filtered for at least 30 deduplicated tags and a fold change (FC) over IgG of ≥2 (normalized total read counts). Regions were considered bound by PPARβ/δ in TAMs if they enrichment sites were observed in at least two out of three TAM samples sequenced. PPARβ/δ binding in MDMs has been described elsewhere [28]. For Figure 2E, PPARβ/δ-occupied genes were identified as genes with a transcription start site close to, or within 50 kb of, an enrichment site. All genomic sequence and gene annotation data was retrieved from Ensembl revision 74.

### Functional annotations and pathway analyses

Functional annotations and pathway analyses were performed using the Ingenuity Pathway Analysis (IPA) application and knowledge database (Qiagen Redwood City, CA, USA). Results were sorted according to p-value of overlap (minimum 0.001) and activation z-scores (≤-2.0 or ≥+2.0) Sequencing data were deposited at EBI ArrayExpress (accession number E-MTAB-3166).

### Statistical analysis of experimental data

Data are presented as the average of replicates (*n* = 3 unless indicated otherwise) with error bars indicating standard deviations and horizontal lines in dot plots representing averages. Comparative data were statistically analyzed by Student's *t*-test (two-sided, equal variance) and results expressed as follows: ns, not significant (*p* ≥ 0.05); *p < 0.05, **p < 0.01 or ***p < 0.001.

### Survival-associated gene expression analysis

Associations between gene expression and relapse-free survival of ovarian cancer patients were analyzed using the web based tool “KM Plotter” (http://kmplot.com/analysis/index.php?p=service&cancer=ovar) [62] with the following settings: ‘auto select best cutoff’, stage: 2+3+4, histology: serous, dataset: TCGA; other settings: default). Logrank Mantel-Cox test (p-values), logrank Hazard Ratio (HR) and median survival times were calculated using the GraphPad Prism software.

## SUPPLEMENTARY MATERIALS FIGURES AND TABLES





## Abbreviations

AA: arachidonic acid
ALA: α-linolenic acid
ANGPTL4: angiopoietin-like 4
ChIP: chromatin immune precipitation
BMDM: marrow-derived macrophage
ChIP-Seq: ChIP sequencing
D10: DMEM with 10% FCS
DHA: docosahexaenoic acid
EPA: eicosapentaenoic acid
FCS: fetal calf serum
15-HETE: 15-hydroxyeicosatetraenoic acid
IPA: Ingenuity Pathway Analysis
6-kPGF_1α_: 6-keto-prostglandin F_1α_
LA: linoleic acid
MDM: monocyte-derived macrophage
NFκB: nuclear factor κB
PDK4: pyruvate dehydrogenase 4
PPAR: peroxisome proliferator-activated receptor
PPARβ/δ: proliferator-activated receptor β/δ
PPRE: PPAR response element
PUFA: polyunsaturated fatty acid
RNA-Seq: RNA sequencing
RT-qPCR: reverse transcriptase quantitative PCR
RXR: retinoid X receptor
TAM: tumor-associated macrophage
XV0: X-VIVO 10 medium without serum
