# Supplementary material for: Deregulation of PPARβ/δ target genes in tumor-associated macrophages by fatty acid ligands in the ovarian cancer microenvironment
Source: Oncotarget. 2015 Apr 15;6(15):13416–33. doi: 10.18632/oncotarget.3826 (PMC4537024; doi:10.18632/oncotarget.3826)
Supplement: Supplementary file 1 [file oncotarget-06-13416-s001.pdf]

Deregulation of PPARβ/δ target genes in tumor-associated macrophages by fatty acid ligands in the ovarian cancer microenvironment

Supplementary Material

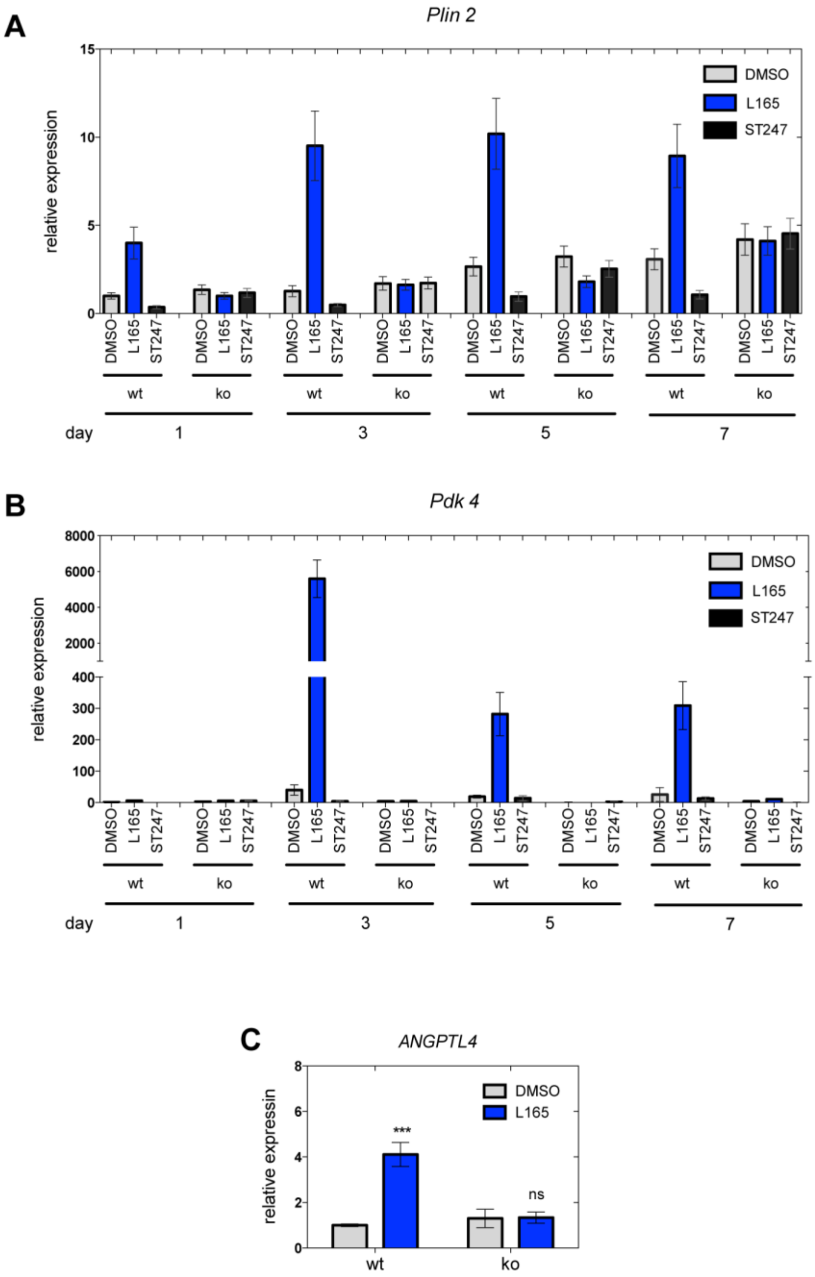

Figure S1. Specificity of L165,041 and St247. (A, B) Mouse bone marrow cells from wt and *Ppard* null mice were differentiated to macrophages in the presence of GM-CSF (as published by Lieber *et al.,* 2015) for 1, 3, 5 or 7 days as indicated in the Figure, followed by 6 h in GM-CSF plus ST247, L165,041 or solvent (DMSO). RNA was analyzed for expression of the PPARβ/δ target genes *PLIN2* (B) and *PDK4* (B) by RT-qPCR as described in Materials and Methods. (C) Thioglycollate-elicited peritoneal macrophages from wt and *Ppard* null mice cultured for 1 day were treated with L165,041 for 6 h as described (Naruhn *et al.,* 2011) and analyzed for *PDK4* expression.

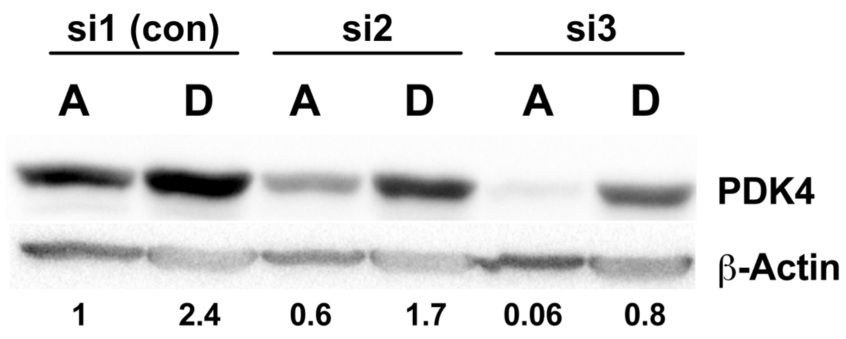

Figure S2. Specificity of the α-PDK4 antibody used for immunoblotting. PDK4 has previously been shown to be induced in detached MCF10A mammary epithelial cells (Grassian *et al.,* 2011), which is reproduced in the immunoblot shown above (A: attached: D: detached). Furthermore, intensity of the PDK4 bands was drastically diminished by pretreatment of the cells with a siRNA against *PDK4* mRNA (si3) compared to a negative control siRNA (si1). si3 is directed against a PDH subunit, which indirectly affects the level of PDK4. Number below the blot represent signal intensities relative to the left-most lane.

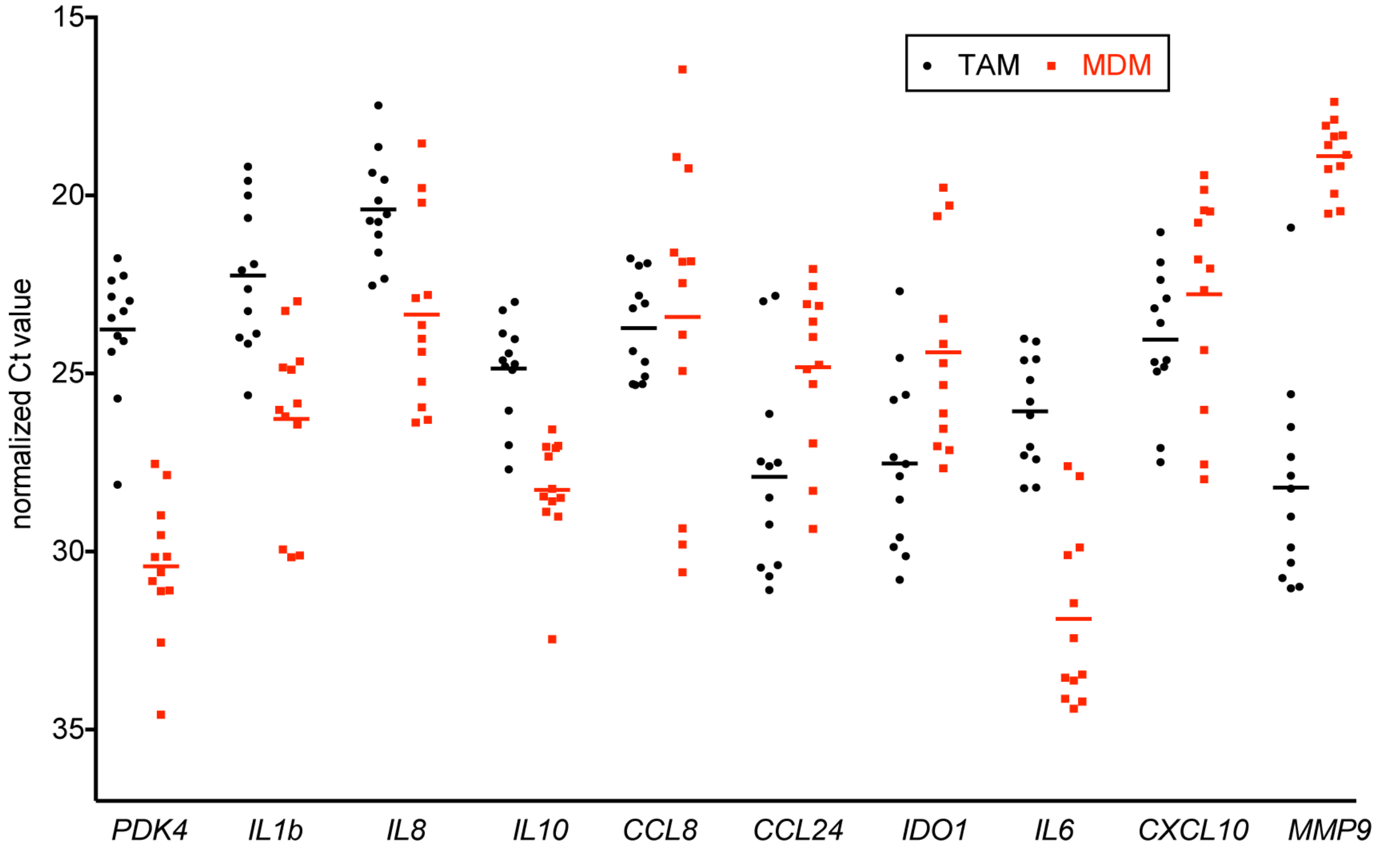

Figure S3. RT-qPCR analysis of PPARβ/δ target gene expression levels in TAMs and MDMs from 17 patients and 12 healthy donors, respectively. Horizontal bars indicate the median. *PDK4* is a canonically regulated gene, all others are inverse target genes.

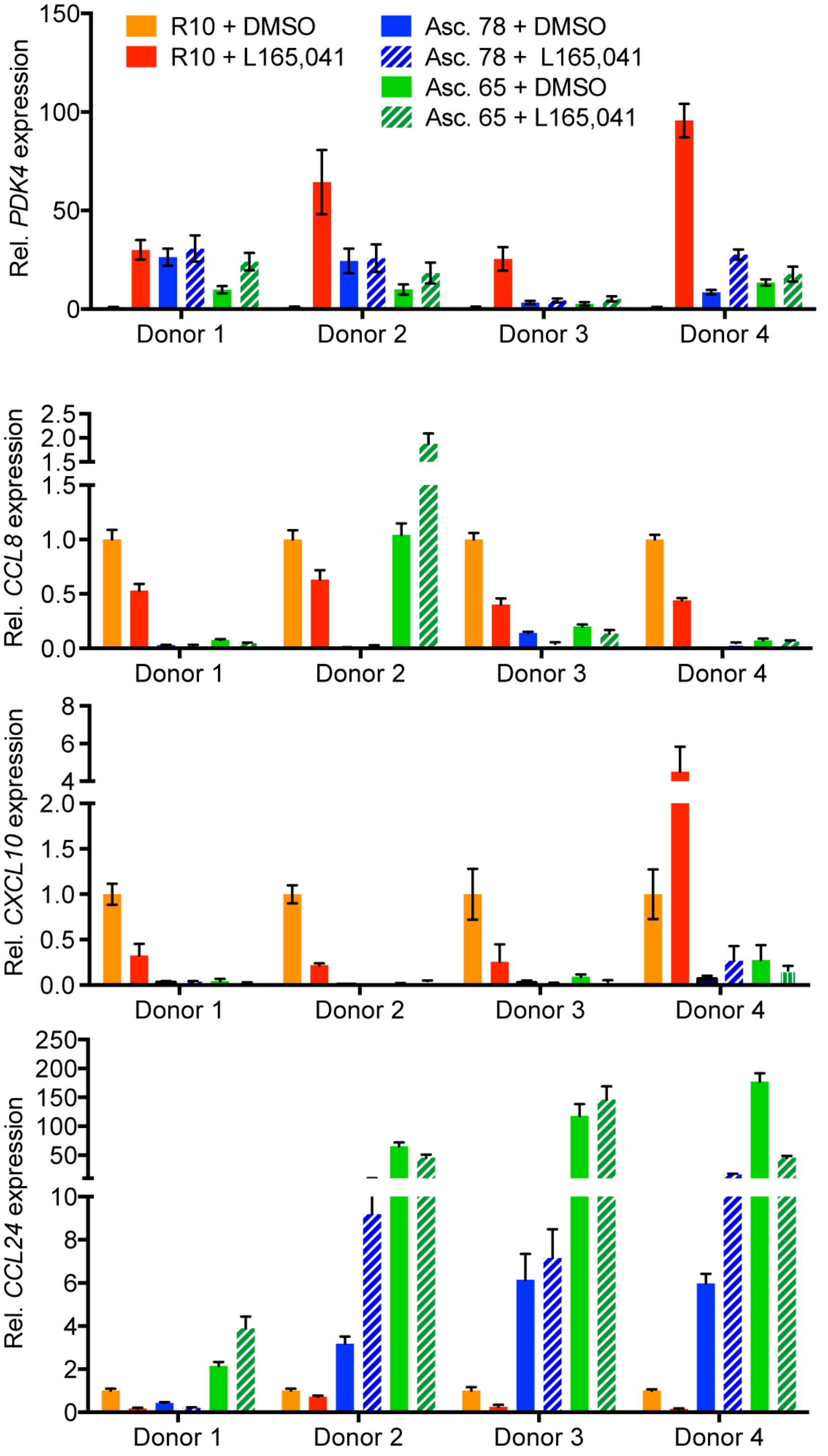

Figure S4 Regulation of inverse target genes by L165,041 in MDMs in normal cell culture medium (red bars) and 2 different ascites samples (blue and green bars) analyzed by RT-qPCR. For comparison, the direct target PPARβ/δ gene *PDK4* is included (top panels). Values indicate expression relative to DMSO/R10-treated cells (1.0) and represent averages of triplicates ± standard deviation.

References

Grassian AR, Metallo CM, Coloff JL, Stephanopoulos G and Brugge JS. Erk regulation of pyruvate dehydrogenase flux through PDK4 modulates cell proliferation. Genes Dev. 2011; 25:1716-1733.

Lieber S, Scheer F, Finkernagel F, Meissner W, Giehl G, Brendel C, Diederich WE, Muller-Brüsselbach S and Müller R. The inverse agonist DG172 triggers a PPARbeta/delta-independent myeloid lineage shift and promotes GM-CSF/IL-4-induced dendritic cell differentiation. Mol. Pharmacol. 2015; 87:162-173.

Naruhn S, Toth PM, Adhikary T, Kaddatz K, Pape V, Dörr S, Klebe G, Müller-Brüsselbach S, Diederich WE and Müller R. High-affinity peroxisome proliferator-activated receptor beta/delta-specific ligands with pure antagonistic or inverse agonistic properties. Mol Pharmacol. 2011; 80:828-838.
